# Amygdalin Reverses Macrophage PANoptosis Induced by Drug-Resistant *Escherichia coli*

**DOI:** 10.4014/jmb.2306.06030

**Published:** 2023-07-28

**Authors:** Xue Yan, Liang Jin, Huifen Zhou, Haofang Wan, Haitong Wan, Jiehong Yang

**Affiliations:** 1 School of Life Sciences, Zhejiang Chinese Medical University, Hangzhou, Zhejiang 310053, P.R. China; 2 School of Basic Medicine, Zhejiang Chinese Medical University, Hangzhou, Zhejiang 310053, P. R. China

**Keywords:** Drug-resistant *E. coli*, amygdalin, PANoptosis, Nrf2

## Abstract

Infectious diseases caused by drug-resistant *Escherichia coli* (*E. coli*) pose a critical concern for medical institutions as they can lead to high morbidity and mortality rates. In this study, amygdalin exhibited anti-inflammatory and antioxidant activities, as well as other potentials. However, whether it could influence the drug-resistant *E. coli*-infected cells remained unanswered. Amygdalin was therefore tested in a cellular model in which human macrophages were exposed to resistant *E. coli*. Apoptosis was measured by flow cytometry and the lactate dehydrogenase (LDH) assay. Western immunoblotting and quantitative reverse-transcription polymerase chain reaction (qRT-PCR) were used to quantify interleukin-18 (IL-18), interleukin-1β (IL-1β), and interleukin-6 (IL-6). The production of reactive oxygen species (ROS) in macrophages was detected by ROS kit. The expression of panapoptotic proteins in macrophages was measured by qRT-PCR and Western immunoblotting. Drug-Resistant *E. coli* inhibited cell viability and enhanced apoptosis in the cellular model. In cells treated with amygdalin, this compound can inhibit cell apoptosis and reduce the expression of pro - inflammatory cytokines such as IL-1β, IL-18 and IL-6. Additionally, it decreases the production of PANoptosis proteins, Furthermore, amygdalin lowered the levels of reactive oxygen species induced by drug-resistant *E. coli*, in cells, demonstrating its antioxidant effects. Amygdalin, a drug with a protective role, alleviated cell damage caused by drug-resistant *E. coli* in human macrophages by inhibiting the PANoptosis signaling pathway.

## Introduction

*Escherichia coli* is one of the most adaptable and pathogenic bacteria, and a cause of various human infections, including gastrointestinal and extraintestinal diseases. It is also a member of the commensal flora in the human and other mammalian intestines [1]. As a result of antibiotic abuse, *E. coli* strains encoding extended-spectrum β-lactamases (ESBL) have emerged, raising concerns about the lack of effective treatment choices for *E. coli* infections and effectively negating the use of whole classes of antibiotics leaving few alternatives [2]. Infections caused by drug-resistant E. coil are a cause of concern for medical establishments due to the high morbidity and mortality rates associated with them [3]. In the case of multidrug-resistant *E. coli* isolates, antibiotic treatment plays a crucial role in clinical practice. However, resistance to other antibiotic counterparts emerges during clinical treatment and poses a significant public health challenge. Therefore, discovery of new drug candidates with unique mechanisms of action is imperative.

Yinhua pinggan granule (YHPGKL), an improved traditional Chinese medicine, contains honeysuckle, licorice, raw bitter almond, and other ingredients [4]. These components exhibit reported anti-infective, antiviral, antioxidant, and anti-apoptotic effects [5]. For example, Guo *et al*. reported that glycyrrhitinic acid, a component of licorice, had a protective effect on multidrug-resistant *Acinetobacter baumannii* (MDR-AB) invasion of A549 cells at appropriate concentrations [6]. Amygdalin, studied in this investigation, is one of the main active compounds of raw bitter almond and has antioxidant, antibacterial, anti-inflammatory, and immunomodulatory activities [7]. Moreover, interest in the pharmaceutical potential of amygdalin has recently been increasing. Pharmacodynamic studies of amygdalin have shown that, along with blocking cancer cell growth and possessing anti-atherosclerotic properties, amygdalin could also relieve inflammation, suppress cough, and eliminate phlegm [8-10]. In a recent report, amygdalin attenuated airway epithelial cell apoptosis, inflammation, and epithelial-mesenchymal transition by inhibiting TLR4/NF-κB signaling in lipopolysaccharide (LPS)-induced cell injury [11]. However, the hypothesis that amygdalin could affect cells infected with drug-resistant bacteria remained to be tested.

The immune system has developed an array of pathways to limit microbial infections and control responses to inflammation. In addition to scorch death downstream of inflammatory vesicle activation, there are several other programmed cell death pathways. PANoptosis is an emerging mechanism that forms due to the crosstalk and coordination between three pathways (*i.e.*, scorch death, apoptosis, and necroptosis) [12, 13]. Reports suggest that the redundant role of cysteine proteases (caspase-8 and caspase-1) and NLRP3 inflammasome is a cross-function of scorch death, apoptosis, and necroptosis molecules [14-16]. The Z-DNA-binding protein 1 (ZBP1), also known as a DNA-dependent activator of interferon regulatory factor (DAI), is known to be a key mediator of NLRP3 inflammatory vesicle activation and PANoptosis apoptosis under certain conditions [17]. Studies have shown that downregulation of ZBP1 reduced total apoptosis of alveolar epithelial cells and ultimately improved acute lung injury [18]. Overall, PANoptosome components are associated with many human illnesses, including auto-inflammatory diseases, neurodegenerative diseases, cancer, microbial infections, and metabolic disorders [19].

Therefore, we investigated the potential protective effect of amygdalin on human macrophages and the mechanism of reversing cell damage induced by drug-resistant *E. coli*.

## Materials and Methods

### Reagents and Materials

Human macrophages were obtained from the Chinese Academy of Sciences (China). Amygdalin (≥ 98% by HPLC, CAS No.: 29883–15–6) was purchased from Chengdu Alfa Biotechnology Co., Ltd. (China). SYBR Premix Ex Taq II Reagent Kit was purchased from Toyobo (China). IL-18, IL-1β, and IL-6 were obtained from Enzyme Marker Biology (China). A Cell Counting Kit-8 (CCK-8) and Hoechst 33258 staining solution were purchased from Beyotime Biotechnology Co., Ltd. (China). A lactate dehydrogenase (LDH) kit, FITC Annexin V apoptosis detection kit, and phosphate-buffered saline (PBS) powder were purchased from Beyotime Biotechnology Co., Ltd.

### Bacterial Cultures and Determination of Minimum Inhibitory Concentration (MIC)

Drug-resistant clinical isolates of *E. coli* used in this study were obtained from the clinical laboratory of Hangzhou First Peoplés Hospital. Isolates were inoculated into Luria-Bertani (LB) broth medium and incubated at 37°C (with shaking at 200 ×*g*) until they reached the logarithmic phase of growth. Bacterial suspensions were diluted with PBS to a cell density of 10^5^ CFU/ml. Based on clinical medication, we selected two types of antibiotics, namely drug-resistant antibiotic cefotaxime sodium (CTX) and sensitive antibiotic tigecycline (TIG), and tested the MIC of both types of antibiotics. The MICs of CTX, TIG, and amygdalin (sterile water configuration) were determined against drug-resistant *E. coli* isolates according to the guidelines of the Clinical and Laboratory Standards Institute (CLSI) 2021-M100 [20]. The tested bacteria were exposed to serial dilutions of the drugs in a 96-well plate, and the absorbance values were measured after an 18 h incubation period.

### Cell Line and Determination of the Drug-Free Concentration

In this study, we utilized human macrophages to test the protective effect of amygdalin. Macrophages were cultured in the RPMI 1640 medium (GIBCO, China) containing 10% fetal bovine serum (BI, FBS, Poncho) and 100 U/ml of penicillin-streptomycin solution (Beyotime, China), and were incubated at 37°C with 5% CO_2_. Log-proliferating-phase cells were transferred to 96-well plates (1 × 10^4^ cells/well). A volume of 100 μg/ml of phorbol-12-myristate-13-acetate (PMA) was added to the wells to induce transformation into THP-1 cells for 48 h, by which time most of the cells were already attached to the wall. After 24 h of incubation, the cell viability was measured at multiple dilutions using the CCK-8 method.

### Co-Culturing of Macrophages and Drug-Resistant E. coil

Macrophages were cultured as described above, and log-proliferating cells were transferred to 6-well plates (5 × 10^5^ cells/well). Subsequently, 100 μg/ml of PMA was added to induce transformation into THP-1 cells for 48 h. Then, the original medium of the orifice plate was replaced with the medium without the penicillin-streptomycin solution. Drug-resistant *E. coli* were used to infect the THP-1 cells according to the optimal multiplicity of infection (MOI=1.5). The THP-1 cells were then incubated in a cell-culture incubator containing 5% CO_2_ for subsequent experiments.

### Examination of the Protective Effect of Amygdalin on Drug-Resistant E. coil-Infected Cells

The LDH method was used to examine the influence of amygdalin on drug-resistant *E. coli*-invaded cells. After macrophages with complete differentiation were dispensed into 24-well plates (5 × 10^4^ cells/well), the optimal MOI amount of drug-resistant *E. coli* and amygdalin was added, and the co-culture was incubated for 18 h. The supernatant was collected and centrifuged at 3,000 × *g* for 5 min, and the absorbance value at 490 nm was measured by an enzyme marker according to the manufacturer’s instructions.

### Detection of Apoptosis Rate

Apoptosis was detected using the Annexin V-FITC Apoptosis Kit (BD, China). Completely differentiated macrophages were co-cultured with drug-resistant *E. coli* and amygdalin in 6-well plates and incubated for 18 h. The supernatant was collected, and the adherent cells were gently trypsinized and collected to detect apoptosis using flow cytometry according to the manufacturer’s instructions. Moreover, the collected supernatant was examined using an inverted microscope for any changes in cell morphology induced by drug-resistant *E. coli* with or without treatment with amygdalin.

### Measurement of Reactive Oxygen Species (ROS)

The ROS was measured using an ROS detection kit (Beyotime). The suspension containing the drug-resistant *E. coli* and amygdalin was co-cultured with macrophage cells for 18 h. The supernatant was then aspirated, and 1 ml of the 1,640-fold diluted probe was added to each well. The mixture was incubated for 30 min protected from light to prevent light-induced auto-oxidation. The supernatant was aspirated, and the cells were collected after trypsin digestion. The intracellular ROS levels were measured by flow cytometry using a dichloro-dihydro-fluorescein diacetate (DCFH-DA) probe as a general oxidative stress indicator.

### Quantitative Reverse-Transcription Polymerase Chain Reaction (qRT-PCR)

For extracting RNA from bacterial cell co-cultures, total RNA was prepared from cells by dispensing 1 ml of Trizol reagent (Fisher CRKP, China) into each well. The RNA was purified by a series of centrifugation cycles after adding the inorganic reagents. The Rever Tra Ace qPCR Kit (Toyobo, China) was used to prepare cDNA using the SYBR Green Real-Time PCR Master Mix (Toyobo, China) on a PCR machine (Quant Studio 12K Flex Real-Time PCR System, Applied Biosystems, China) following the instructions of the kit provider. GAPDH was used as an endogenous control and normalized. The relative expression of genes was calculated using the 2^-ΔΔCt^ method, where Ct is the cycle threshold. The primer sequences were shown in Table 1.

### Western Immunoblotting

The cell samples of each group were collected, and the total proteins were extracted and stored at 4°C. The proteins were separated by electrophoresis and transferred onto membranes, which were then incubated overnight with rabbit anti-human antibodies for β-actin, Nrf-2, HO-1, SOD1, IL-6, IL-1β, IL-18, caspase-1, caspase-3, caspase-7, caspase-8, NLRP3, GSDMD, p-MLKL, p-PIPK3, and IGF2BP1 at 4°C. The membranes were then washed with Tris-HCl-Tween (TBST) for 10 min and incubated with an anti-rabbit IgG secondary antibody (Thermo Pierce, China) for 1 h at room temperature. Finally, the blots were visualized by a gel imager (Bio-Red, Shanghai, China) using an enhanced chemiluminescence (ECL) agent. Grayscale values were analyzed using ImageJ software (https://imagej.net/). β-Actin was used as an endogenous control and normalized.

### Statistical Analysis

All data were expressed as mean ± standard deviation (SD). One-way ANOVA (analysis of variance) was used to assess the differences between groups, followed by SPSS multiple comparison tests. Significant differences were presented as significant (*p* < 0.05) or highly significant (*p* < 0.01).

## Results

### Analysis of Antibacterial and Cytotoxicity Activities

**Fig. 1:**
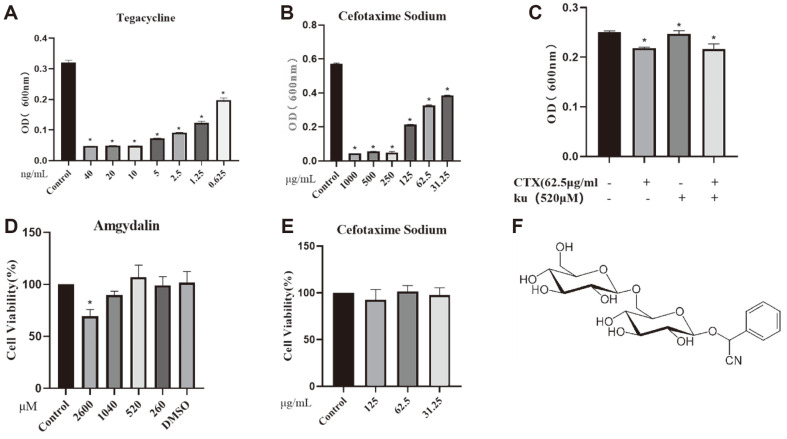
Bacterial drug resistance and cytotoxicity analysis. Add different concentrations of antibiotics to *Escherichia coli* for co cultivation for 24 h to detect their inhibitory effect, determine the minimum inhibitory concentration of antibiotics (**A, B**), Bacteriostatic effect of different concentrations of amygdalin alone or in combination with antibiotics (**C**), CCK-8 assay for cytotoxicity of amygdalin and antibiotics on macrophage cells (**D, E**), Molecular formula of amygdalin (**F**). *P < 0.05 vs. the control group.

Based on clinical medication, we selected two types of antibiotics for screening. We evaluated the antibacterial activity of amygdalin and the antibiotics CTX and TIG against the drug-resistant *E. coli*. The data revealed that the tested *E. coli* isolate was susceptible to TIG (Fig. 1A), while the CTX MIC was 250 μg/ml (Fig. 1B). These antibiotics were selected for the control experiments. The results also indicated that amygdalin had no measurable antibacterial effect at the maximum solubility of 520 μM (Fig. 1C). We further analyzed the cytotoxicity of amygdalin on human macrophage cells, which were treated with CTX and amygdalin for 24 h, and the cytotoxicity was evaluated using the CCK-8 kit. As shown in Figs. 1D-1E, amygdalin (520 μM) and CTX (62.5 μg/ml) had no toxic effect on the tested cells.

### Effect of Amygdalin on the Cell Viability of *E. coli*-Infected Macrophages

**Fig. 2:**
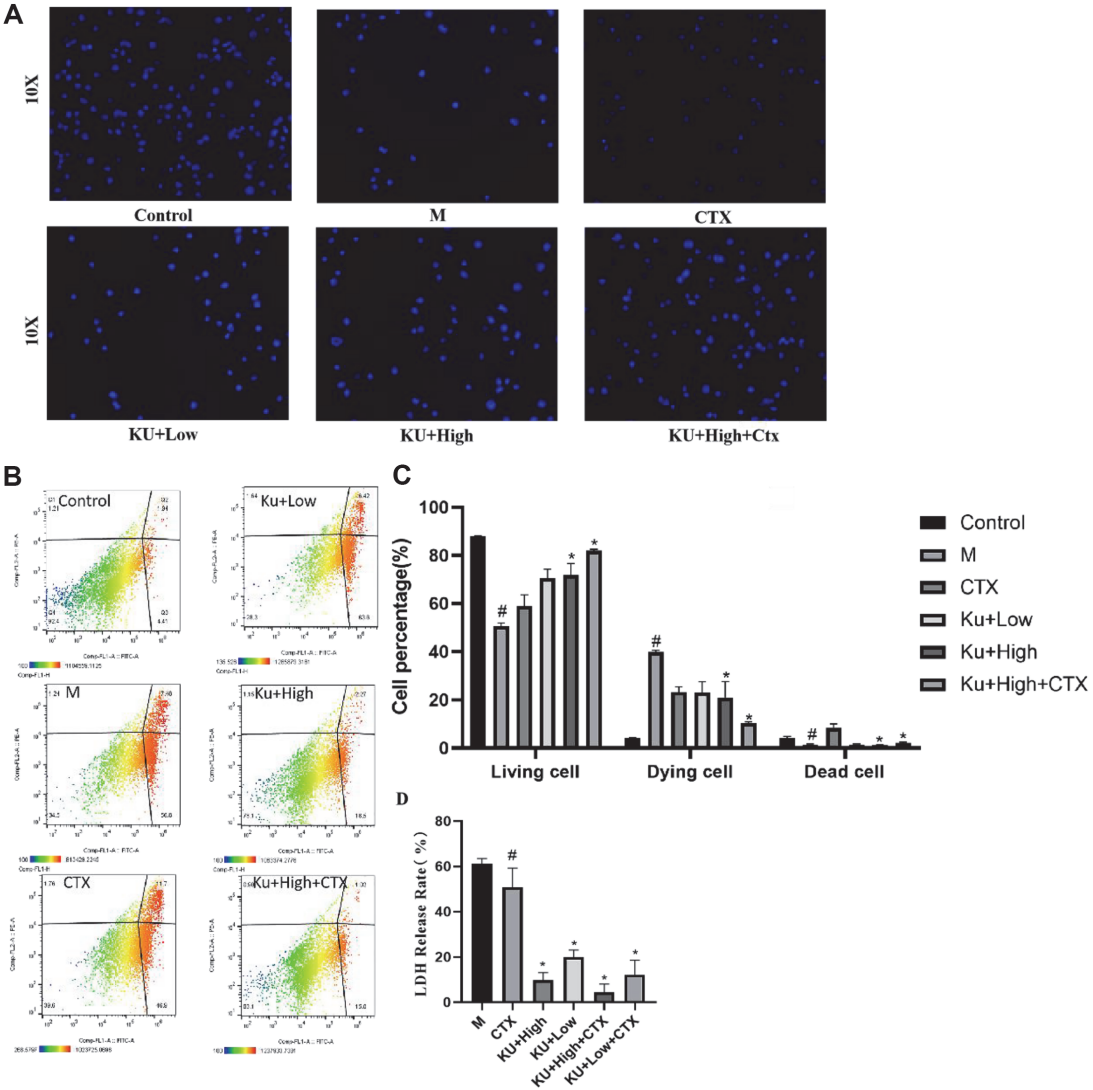
Effect of amygdalin on drug-resistant *E. coli*-infected macrophages. To test the protective effect of amygdalin on cell apoptosis, Cell morphology was observed by an inverted microscope (**A**). Amygdalin reduced the proportion of cell death and increased the percentage of viable cells in drug-resistant *E. coli* infestation and had a synergistic effect with CTX (**B, C**). LDH assays were used for further verification (**D**). ^#^*P* < 0.05 vs. the control group; *P < 0.05 vs. the drugresistant *E. coli*-infected group.

To investigate whether amygdalin could protect human macrophage cells from *E. coli* damage, macrophages were infected with drug-resistant *E. coli* and co-cultured with both amygdalin and CTX for 18 h. We conducted multiple verifications and used the CTX group as a control to further observe whether amygdalin can improve the efficacy of antibiotic resistance in combination with CTX, providing new ideas for clinical medication. We used Hoechst 33258 staining agent to stain living cells and observed their cell morphology under an inverted microscope (Fig. 2A). In addition, the Annexin V- FITC and PI staining results showed that amygdalin reduced the proportion of cell death and increased the percentage of viable cells in drug-resistant *E. coli* infestation and had a synergistic effect with CTX (Figs. 2B and 2C). In addition, the LDH assays also confirmed the same finding as the flow cytometry results revealed (Fig. 2D).

### Amygdalin Decreased Inflammatory Factors in Drug-Resistant *E. coli*-Infected Cells

**Fig. 3:**
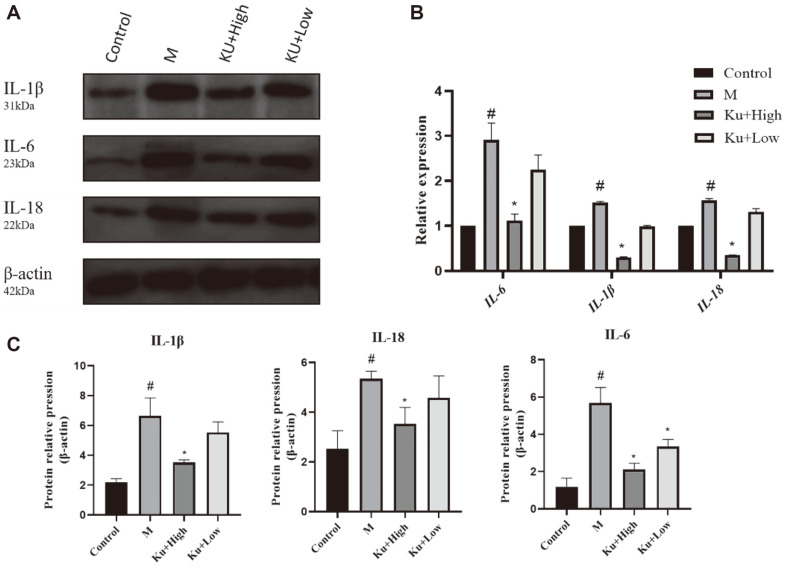
Effect of amygdalin on the production of pro-inflammatory factors by macrophage cells infected with *E. coli*. Effect of amygdalin on the production of pro-inflammatory factors by macrophage cells infected with *E. coli*. We examined the effect of amygdalin on the pro-inflammatory cytokines induced by drug-resistant *E. coli* using WB and qRT-PCR (**A, C**). The PCR results further supported this interpretation (**B**). ^#^*P* < 0.05 vs. the control group; *P < 0.05 vs. the drug-resistant *E. coli*-infected group.

To detect the effect of KU on pro-inflammatory factors in drug-resistant *E. coli*- infected cells, we also examined the effect of amygdalin on the pro-inflammatory cytokines induced by drug-resistant *E. coli* using WB and qRT-PCR. As shown in Figs. 3A-3C, the expression level of inflammatory factors in the drug-resistant *E. coli* infection group was significantly higher than that in the control group. Under amygdalin treatment, the expression level of pro-inflammatory factors was significantly reduced and had a synergistic effect with CTX. These results suggested that amygdalin reduces the expression of inflammatory factors in cells.

### Amygdalin Reversed PANoptosis Induced by Drug-Resistant *E. coli*

**Fig. 4:**
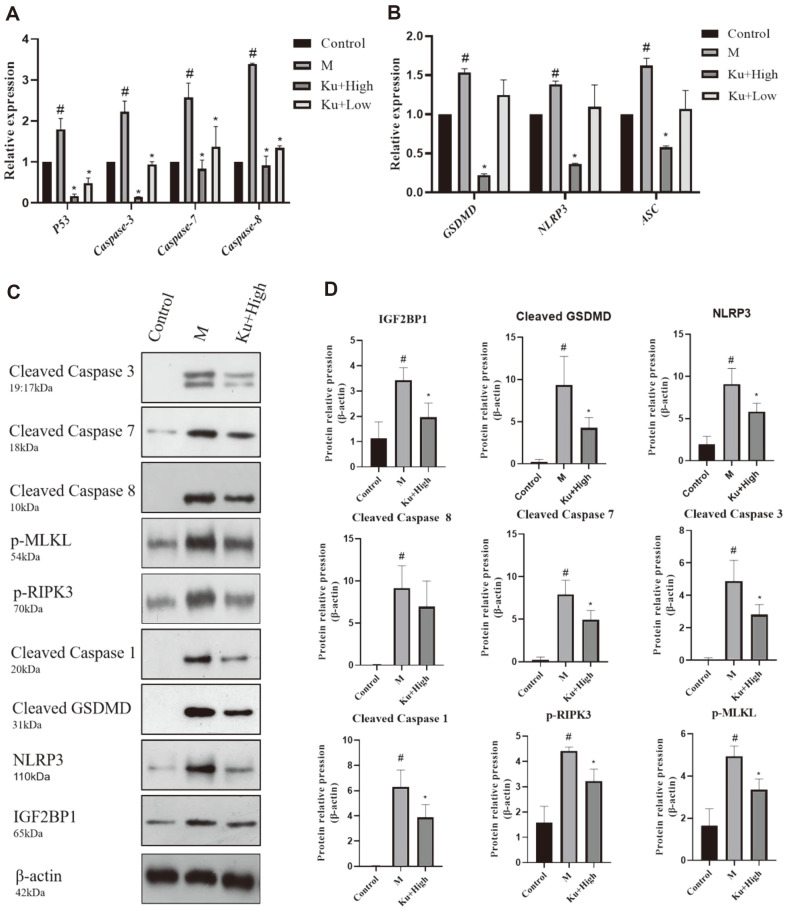
Effect of amygdalin on the induction of PANoptosis by drug-resistant *E. coli*-infected cells. The mRNA expression of caspase-3, caspase-7, and caspase-8 and the GSDMD and NLRP3 scorch genes was assessed by RT-PCR (**A, B**). The protein expression of necroptotic apoptotic proteins p-MLKL, p-PIPK3, and IGF2BP1 among all groups was analyzed by Western blot, and a statistical result was calculated (**C, D**). ^#^*P* < 0.05 vs. the control group; *P < 0.05 vs. the drug-resistant *E. coli*infected group.

To confirm that amygdalin reduces cell damage through PANoptosis, we examined the apoptotic proteins caspase-3, caspase-7, and caspase-8, the necroptotic apoptotic proteins p-MLKL and p-PIPK3, the scorch death proteins GSDMD and NLRP3, and caspase-1 in *E. coli*-infected macrophage cells. As shown in Figs. 4A-4B, the mRNA expression of the *E. coli*-induced apoptosis genes and pleocytosis genes was significantly increased compared with the control group. This trend was drastically reversed after the amygdalin addition. We analyzed the expression of pan-apoptotic vesicle proteins by WB. In this study, the expression of proteins was higher in the drug-resistant *E. coli*-infected group compared with the control group. The expression of all tested proteins was significantly decreased upon treatment with amygdalin (Figs. 4C and 4D).

### Effect of Amygdalin on the Production of ROS by Macrophages

**Fig. 5:**
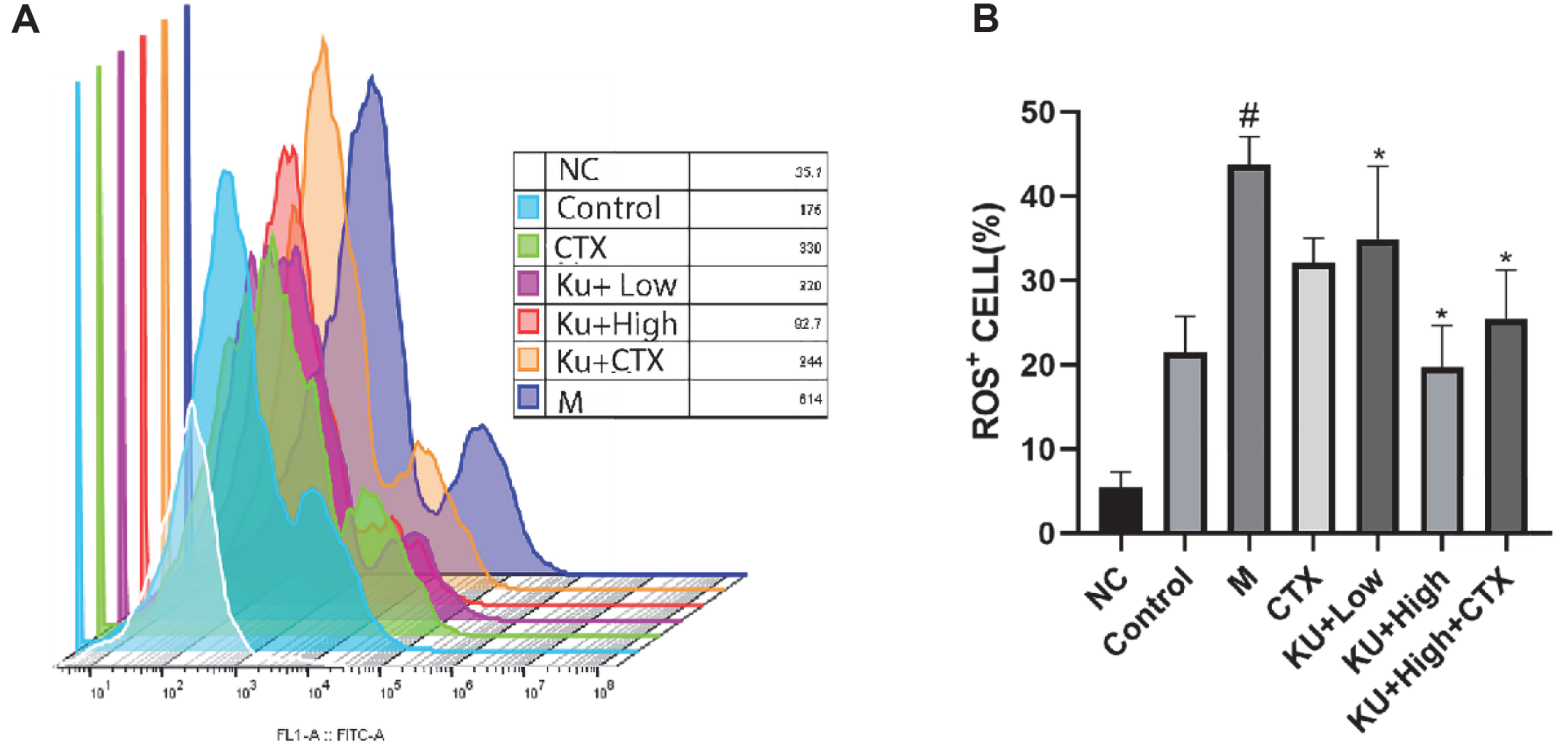
Effect of amygdalin on the level of ROS released from macrophages induced by drug-resistant *E. coli*. ROS levels were measured using flow cytometry (**A, B**). ^#^*P* < 0.05 vs. the control group; *P < 0.05 vs. the drug-resistant *E. coli*infected group.

We examined the ability of amygdalin to regulate the production of excessive ROS by macrophage cells infected by drug-resistant *E. coli*. The flow cytometry results revealed a significant increase in ROS content in macrophage cells after *E. coli* stimulation compared to the control group. Meanwhile, amygdalin exhibited antioxidant effects, as evidenced by a dose-dependent decrease in ROS production (Figs. 5A and 5B).

### Effect of Amygdalin on Oxidative Stress Damage Caused by Drug-Resistant *E. coli*

**Fig. 6:**
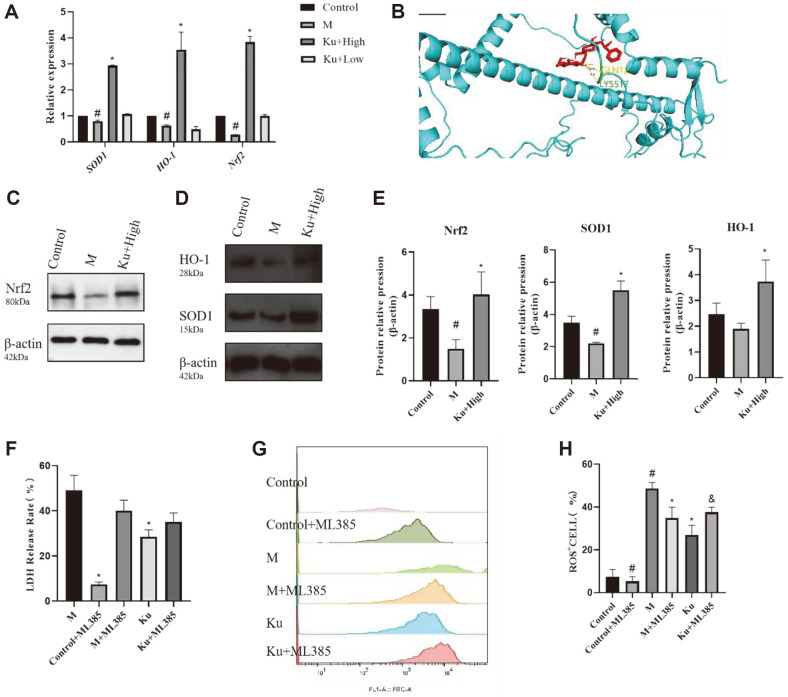
Effect of amygdalin on oxidative stress induced on macrophages by the drug-resistant *E. coli* isolate. The real-time expression of SOD1, Nrf2 and HO-1 mRNA was detected by RT-PCR, as shown in (**A**). Detection of the target of amygdalin and Nrf2 by Macromolecular docking. (**B**) Representative results of the SOD1, Nrf2 and HO-1 protein expression analyzed by Western immunoblotting (C-E). The findings were validated using the Nrf-2 inhibitor ML385 (F-H). ^#^*P* < 0.05 vs. the control group; *P < 0.05 vs. the drug-resistant *E. coli*-infected group; and ^&^*P* < 0.05 vs. the KU group.

Nrf-2 is a powerful antioxidant transcription factor that activates heme oxygenase-1 (HO-1) and superoxide dismutase (SOD1) to promote cellular antioxidant activity [21]. Here, we confirmed that amygdalin could activate the Nrf-2 pathway by WB and RT-PCR analyses. As shown in Fig. 6A, Nrf-2, HO-1 and SOD1 expression was inhibited by the drug-resistant *E. coli* at both the transcriptional and translational levels, but amygdalin could restore its expression. The results of WB also confirm this result (Figs. 6C-6E). As shown in Fig. 6B, amygdalin and Nrf2 targets have similar affinity, and the space formed after docking is stable. Therefore, amygdalin can activate the Nrf2 signaling pathway to inhibit ROS production. For validation, the Nrf-2 inhibitor (ML385) was used in the experiment. The LDH assay results further confirmed that amygdalin repaired the oxidative stress damage in the drug-resistant *E. coli*-infected cells by activating the Nrf-2 pathway (Fig. 6F). Flow cytometry detection of ROS release further validates this result (Figs. 6G and 6H).

## Discussion

*Escherichia coli* is a member of the *Enterobacteriaceae* family and is considered the most prevalent commensal bacteria in the gastrointestinal tract of humans and warm-blooded animals. *E. coli* can establish symbiotic associations with their host organisms, which could be mutually beneficial. On the other hand, the pathogenicity of *E. coli* is well known, and the bacterium is one of the most serious pathogens [22]. Carbapenems are the first-line therapy against problematic *E. coli* infections [23]. However, due to the increased emergence of drug-resistant *E. coli* worldwide, the use of carbapenems is inevitably rising. This situation has led to the overuse of carbapenems and posed genuine concerns about the development of carbapenem-resistant strains. Antibiotics are chemical structures of different classes and different modes of action. Likewise, bacteria have developed various mechanisms to resist the harmful effects of antibiotics [24]. These resistance mechanisms include the inactivation of antibiotics, modification/alteration of antibiotic targets, prevention of antibiotic access, and antibiotic efflux pumps. When encountered by external threats (especially antibiotics), bacteria can employ multiple defense mechanisms simultaneously to counteract these threats and ensure their survival [25]. Therefore, the search for novel drug alternatives to antibiotics is accelerating. For this reason, there has been a renewed focus on herbal ingredients recently, with many monomers showing significant potency. A previous study reported that glycyrrhizic acid could reverse cellular damage induced by drug-resistant *Klebsiella pneumoniae*. The inflammation was reduced to some extent by glycyrrhizic acid treatment. Glycyrrhizic acid exerted oxidative homeostasis by activating the Nrf-2 signaling pathway to produce antioxidant proteins [26]. Recent studies also reported that lipopolysaccharide (LPS) enhanced apoptosis and promoted the production of inflammatory factors, such as TNF-α, IL-6, and IL-18 by the lung epithelial BEAS-2B cells, while amygdalin pretreatment counteracted the effect of LPS. Amygdalin attenuated airway epithelial cell apoptosis, inflammation, and epithelial-mesenchymal transition by inhibiting the TLR4/NF-κB signaling pathway in cough variant asthma [27]. In the present study, we report that amygdalin could reverse the macrophage PANoptosis induced by drug-resistant *E. coli*. The PANoptosis was reduced to some extent by the intervention of amygdalin.

ROS are an essential component of the antimicrobial activity of macrophages [28, 29]. Generating oxidative stress in the pathogen-containing phagosomes is crucial for antimicrobial immunity [30-32]. In addition, there is a growing body of evidence that tightly controlled elevation in cellular ROS levels can have beneficial effects on cells [33, 34]. The primary role of ROS generated by macrophages is to inactivate phagocytosed bacteria through an oxidative burst. Recognition of invading bacteria initiates rapid and robust generation of ROS into the extracellular space and the lumen of the phagosome [29, 35-38]. However, acting in another role, excess ROS can induce cytotoxicity and trigger many forms of cell death [32, 39]. For example, inhibition of methylenetetrahydrofolate dehydrogenase 2 significantly increased ROS levels and induced apoptosis [40]. Moreover, ROS-inducing drugs can bind to iron, increase ROS cellular levels, and subsequently trigger pyrosis in melanoma cells [41]. Studies have shown that amygdalin could effectively reduce ROS levels in HBZY-1 cells induced by high sugar stress. Amygdalin has also exhibited the potential to reduce oxidative stress in the renal tissue of diabetic nephropathy rats. Long-term treatment with amygdalin reduced inflammation by downregulating the expression of cytokines, including IL-2 and IFN-γ, in the renal tissues of rats with diabetic nephropathy [42]. In our study, the levels of inflammatory cytokines and ROS produced by macrophage cells were significantly elevated due to the infection by drug-resistant *E. coli*. Amygdalin treatment of infected macrophage cells could counteract the cellular damage caused by *E. coli*. To find out how amygdalin reduced oxidative stress in infected cells, we further investigated the effect of amygdalin on Nrf-2, a well-known transcription factor for antioxidant-regulated responses. Nrf-2 can activate HO-1 and SOD and thereby exerts powerful antioxidant effects [21]. Data in this study indicated that amygdalin reversed the inhibition of Nrf-2 activity by drug-resistant *E. coli*, thus increasing the antioxidant proteins and decreasing the inflammatory cytokines. This finding suggests that amygdalin could depend on the Nrf-2 signaling pathway to protect cells from drug-resistant *E. coli*-induced damage.

It is evident that cell PANoptosis is dependent on dead cell apoptosis, pleocytosis, and necrosis [43]. Infected cell-induced apoptosis and the release of IL-1β and IL-18 by activating complex vesicles (composed of caspase-3/ -7 activated by RIPK, caspase-8, ASC, and NLRP3) promote GSDMD phosphorylation and lysis via MLKL [15]. It has been reported that cysteine desulfatase (NFS1) deficiency operated synergistically with oxaliplatin to trigger PANoptosis (apoptosis, necroptosis, and cytosolic scorching) and consequently increase the level of intracellular ROS [44]. Insulin-like growth factor 2 mRNA-binding protein 1 (IGF2BP1) is a key regulator of mRNA metabolism and transport during the development of organisms. Recent studies reported that IGF2BP1 was aberrantly expressed in a wide range of tumors, including liver, lung, colon, ovarian, and breast cancers [45]. Significantly higher IGF2BP1 levels were observed by western blotting (WB) analysis in cells that had been infiltrated by bacteria, while the expression was significantly reduced by the use of amygdalin. In the present study, we reveal that amygdalin demonstrated an inhibitory effect on drug-resistant *E. coli*-induced PANoptosis. Our findings showed that the drug-resistant *E. coli*-infected macrophages produced the apoptotic proteins caspase-3, caspase-7, and caspase-8, necroptotic apoptotic proteins p-MLKL and p-PIPK3, and focal proteins GSDMD, NLRP3, and caspase-1. The levels of these proteins decreased significantly by amygdalin treatment, suggesting that amygdalin could protect cells from bacterial damage by inhibiting the macrophage pan-apoptotic pathway.

However, this study has a few limitations. Firstly, due to biosafety restrictions, we could only verify these findings at the cellular level and could not perform the relevant animal studies. Secondly, although amygdalin could inhibit drug-resistant *E. coli*-induced inflammation and oxidative stress, we could only use an Nrf-2 inhibitor to validate this conclusion. Lastly, the interrelationships between cellular antioxidant signaling pathways and the mechanisms of interactions were very complex. Therefore, further studies on other signaling pathways are needed.

## Conclusion

**Fig. 7:**
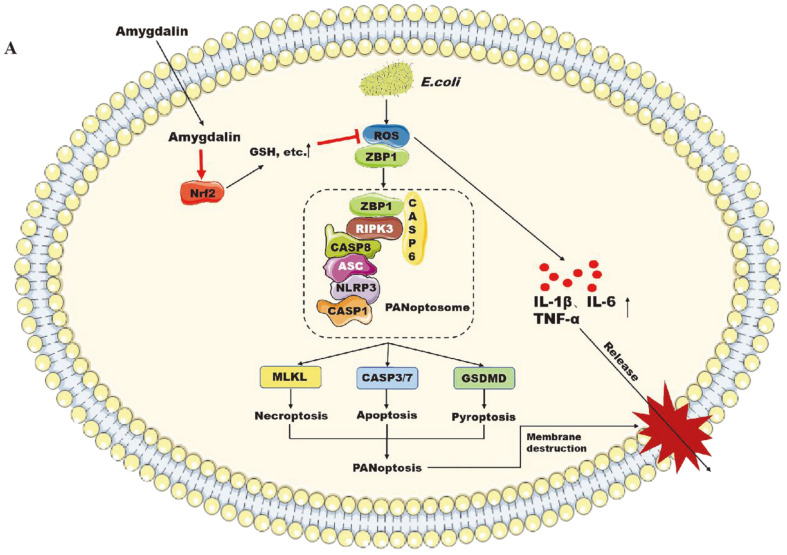
Schematically, drug-resistant *E. coli* could induce excessive ROS production by macrophages to promote the expression of inflammatory factors and could enhance a significant increase in the levels of pan-apoptotic proteins leading to cell death. In contrast, amygdalin could inhibit this process by suppressing the generation of ROS and the expression of pan-apoptotic proteins.

In this study, we report that amygdalin could protect human macrophage cells from drug-resistant *E. coli* infestation by inhibiting cell PANoptosis. As shown in Fig. 7, this is the first report on amygdalin attenuation of the cell damage caused by drug-resistant *E. coli* through the activation of the pan-apoptotic signaling pathway.

## Figures and Tables

**Table 1:** The primer sequences for qRT-PCR.

Gene	Forward (5'–3')	Reverse (5'–3')
*GAPDH*	GGAGAAGGCTGGGGCTCAT	TGATGGCATGGACTGTGGTC
*P53*	CCCCTCCTGGCCCCTGTCATCTTC	GCAGCGCCTCACAACCTCCGTCAT
*NLRP3*	AAGGCCGACACCTTGATATG	CCGAATGTTACAGCCAGGAT
*IL—1β*	CTGAGCTCGCCAGTGAAATG	TGTCCATGGCCACAACAACT
*IL-18*	TGGCTGCTGAACCAGTAGAA	ATAGAGGCCGATTTCCTTGG
*Caspase-3*	AACATGCCCAAGGAGGAAGA	GGCTGTTCACCAATCCATGA
*GSDMD*	AGCCAGAAGAAGACGGTCA	TCCAAGTCAGAGTCAATAACCA
*ASC*	CTGACGGATGAGCAGTACCA	CAGGATGATTTGGTGGGATT
*Caspase-7*	CGGTCCTCGTTTGTACCGTC	CGCCCATACCTGTCACTTTATCA
*Caspase-8*	TTTCTGCCTACAGGGTCATGC	GCTGCTTCTCTCTTTGCTGAA
*HO-1*	AAGACTGCGTTCCTGCTCAAC	AAAGCCCTACAGCAACTGTCG
*SOD1*	GGTGGGCCAAAGGATGAAGAG	CCACAAGCCAAACGACTTCC
*IL-6*	CCACTCACCTCTTCAGAAC	CTTTGCTGCTTTCACACAT
*Nrf-2*	TCAGCGACGGAAAGAGTATGA	CCACTGGTTTCTGACTGGATGT
